# Exploring if Longitudinal Changes on PET Imaging Can Serve as a Biomarker for Stiff Person Syndrome Spectrum Disorders

**DOI:** 10.1002/acn3.70145

**Published:** 2025-08-07

**Authors:** Munther M. Queisi, Samantha N. Roman, Mohammad S. Sadaghiani, Lilja B. Solnes, Scott D. Newsome

**Affiliations:** ^1^ Department of Neurology Johns Hopkins University School of Medicine Baltimore Maryland USA; ^2^ Department of Radiology Johns Hopkins University School of Medicine Baltimore Maryland USA

**Keywords:** classic SPS, PET, SPS, SPS plus, Stiff Person Syndrome

## Abstract

**Objective:**

To identify metabolic patterns in the brain and musculoskeletal system of stiff person syndrome spectrum disorders (SPSD) patients over time using PET imaging and evaluate the impact of immune therapy on metabolic activity as a surrogate for treatment response.

**Methods:**

This observational study at the Johns Hopkins SPS Center of Excellence included adults (≥ 18 years) diagnosed with classic SPS, partial SPS, SPS plus, or PERM, who were treated from 2009 to 2023. Participants underwent at least two whole‐body and/or dedicated brain PET scans. Brain PET analysis utilized NeuroQ v3.8 software to generate standardized *Z*‐scores for 47 distinct brain regions, assessed by expert nuclear medicine radiologists. Patient demographics, antibody types, immune therapies, and symptomatic treatments were assessed.

**Results:**

Eighteen patients met inclusion criteria (10 SPS plus, 6 classic SPS, 2 PERM), with a mean age of 50.7 (SD 8.5) years; 77.8% were female and 50.0% were Black/African American. Hypermetabolic activity and hypometabolism were both seen over time in the brain and the musculoskeletal system. Two patients starting immune therapy in between PET scans demonstrated improvement in brain but not body PET findings.

**Interpretation:**

The study findings indicate that PET imaging abnormalities are present over time and appear to have regional patterns that are common among phenotypes. Additionally, starting immune therapy between scans appears to correlate with stabilization or improvement in brain PET abnormalities, though this was less clear in body PET scans. PET scans have the potential to be an imaging biomarker in SPSD, but future studies are needed to validate this.

## 1 Introduction

Stiff Person Syndrome Spectrum Disorders (SPSD) are rare, complex, and progressive neuroimmunological disorders typically characterized by rigidity and painful spasms, which can severely impact mobility and quality of life [1, 2, 3, 4]. SPSD encompass a range of conditions with overlapping features, thus presenting a unique set of challenges for healthcare providers [2]. The variability in symptoms and clinical presentations makes SPSD challenging to diagnose, often leading to delays in appropriate treatment [3, 5]. The lack of definitive diagnostic criteria further complicates the clinical landscape, necessitating a nuanced understanding of the disorder [6].

There are several phenotypes that currently fall under the spectrum of SPSD which have unique symptoms and presentations: classic SPS, partial SPS, SPS plus, and progressive encephalomyelitis with rigidity and myoclonus (PERM) [7]. Classic SPS is characterized by painful muscle spasms and rigidity affecting the trunk and lower more than the upper extremities [8]. Cases limited to a single limb or torso are diagnosed as partial SPS or stiff limb syndrome. Patients with SPS plus have classic SPS features alongside cerebellar and/or brainstem deficits. PERM presents with encephalopathy, musculoskeletal rigidity, and myoclonus. Classic SPS is the most common phenotype, seen in approximately 70% of patients, while SPS plus accounts for approximately 12%–30% [2, 6, 9, 10]. The true prevalence of partial SPS and PERM is unknown; however, they are much less common than the aforementioned phenotypes. SPSD are most often associated with anti‐glutamic acid decarboxylase 65 (anti‐GAD65) autoantibodies, and less commonly, anti‐glycine and anti‐amphiphysin antibodies [11].

In a small minority of patients (≤ 5%), especially those with amphiphysin antibodies, SPSD may occur as a paraneoplastic syndrome and can be the first clinical manifestation of an underlying malignancy [9, 12, 13]. As such, a whole‐body fluorodeoxyglucose‐positron emission tomography/computed tomography (FDG‐PET) scan is often obtained as part of a paraneoplastic workup in individuals with SPSD. Prior work by our group highlighted abnormalities seen on both brain and body PET scans among patients with SPSD. Particularly, increased FDG uptake in the skeletal muscles was seen in 63% of the SPSD cohort, and the hypermetabolic muscles correlated with clinical involvement in 42% of cases at the time of the PET scans. Additionally, in the patients who had available electromyography (EMG) for review, 62% had findings on EMG that correlated with abnormal regions on PET scans. This prior study also demonstrated abnormal FDG uptake in the brain associated with SPSD, including brainstem hypermetabolism and various hypometabolic thalamic and cortical regions (frontal, temporal, and occipital) [14, 15].

Given the heterogeneity of SPSD, clinicians often rely on a combination of clinical presentation, ancillary testing, and imaging studies to reach a diagnosis [9, 16]. Furthermore, once a diagnosis is made, evidence is lacking regarding biomarkers or parameters that can be used to prognosticate or assess response to therapy over time. PET represents a potential diagnostic biomarker for SPSD, as abnormal brain and/or skeletal muscle FDG uptake may be supportive of an SPSD diagnosis in the appropriate clinical context [2, 6, 12]. Given the limited data available on PET in SPSD, it remains unclear if abnormalities in the brain and skeletal muscle persist over time or change in the context of treatment with immune therapies and may be useful as a tool to assess treatment response.

The objective of this study was to identify any regional or diffuse patterns of hypermetabolic and/or hypometabolic activity within the brain and musculoskeletal system for SPSD patients longitudinally and to determine if abnormalities correlate with stiffness and spasms on clinical examination. Further, we aimed to evaluate how these abnormalities, if present, might change with the addition of immune therapy and thus could represent a potential biomarker to monitor treatment response.

## 2 Methods

### 2.1 Standard Protocol Approvals, Registrations, and Patient Consents

All participants provided written informed consent as part of our longitudinal SPS study, which was approved by the Johns Hopkins Institutional Review Board.

### 2.2 Study Design and Population

This retrospective study was performed as part of an ongoing observational study at the Johns Hopkins SPS Center of Excellence. Adults (≥ 18 years old) with a diagnosis of SPSD (classic SPS, partial SPS, SPS plus, or PERM) who were treated at the Johns Hopkins SPS Center between the years 2009 and 2023 and had at least two whole‐body (skull base to mid‐thigh) and/or two dedicated brain PET scans documented in their medical records, with source images of sufficient quality to facilitate comparison, were included. Patients were excluded if they had low‐quality PET scans that could not be used for comparison. Patients with autoimmune pure cerebellar ataxia, refractory autoimmune epilepsy, and limbic encephalitis were excluded to ensure integrity and minimize confounding variables, as these conditions in isolation are not thought to be part of SPSD.

### 2.3 Clinical Characteristics

The SPSD diagnosis was determined by an SPS expert (SDN) at Johns Hopkins through a combination of clinical and paraclinical assessments. Demographics (age at time of symptom onset, sex, race, ethnicity) and clinical characteristics (SPSD phenotype, antibody seropositivity, including but not limited to GAD65 antibody, and their corresponding titers) were obtained from the Johns Hopkins SPS longitudinal database and, when necessary, the medical records. An enzyme‐linked immunosorbent (ELISA) assay was used for the serum GAD65 antibody and radioimmunoassay (RIA) for the CSF GAD65 antibody testing. Relevant physical examination findings (e.g., location of abnormal tone/rigidity, presence of abnormalities localizing to the brainstem and/or cerebellum), modified Rankin score (mRS; ordinal scale from 0; asymptomatic to 6; death), symptomatic medications, immune therapies, and timed 25‐foot walk were recorded from the clinic visit nearest to the date of each PET scan. All but two patients had a clinical visit within 90 days of their initial PET scan. Similarly, all but two were receiving symptomatic treatments at the time of imaging, with most likely having received their therapies on the day of the PET scan.

### 2.4 PET Analysis

The analysis of brain PET scans was conducted using NeuroQ v3.8 software, which provides quantitative analysis across 47 distinct brain regions compared to normative data gathered from healthy adults without neuropsychiatric or neurodegenerative diseases. NeuroQ generates standardized *Z*‐scores for each brain region, with scores above zero indicating hypermetabolism and scores below zero indicating hypometabolism. A *Z*‐score with a standard deviation greater than 1.65 was classified as significantly hypermetabolic, while scores below −1.65 were deemed significantly hypometabolic, similar to prior studies. The clinical radiology report for these scans was also reviewed, and abnormalities were compared to NeuroQ findings for discrepancies.

For the evaluation of body PET scans, an expert nuclear medicine radiologist (MSS) reviewed the studies on a Miranda XD3 workstation. The radiologist was aware of an SPSD diagnosis but was blinded to the demographics, clinical presentations, phenotype, examination findings, and immunological profiles of the participants to eliminate interpretation bias. The radiologist assessed the presence and patterns of abnormal metabolism in skeletal muscle for all scans available, providing qualitative data on the muscles with abnormal uptake as well as grading the abnormality as mild, moderate, or marked. Although whole‐body PET imaging is traditionally acquired from the skull base to the mid‐thighs, in some cases where the field of view extended further, the radiologist was able to comment on the area below the thighs down to the distal lower legs. This review was then compared to the clinical radiology report findings for discrepancies. Additionally, PET imaging was also performed at the time of SPS diagnosis, primarily to screen for underlying malignancy, as well as in patients with a history of cancer or those presenting with atypical features or rapid clinical progression.

### 2.5 Statistical Analyses

Summary statistics were completed for patient demographics and clinical characteristics. Semi‐quantitative NeuroQ data were used to create heatmaps, where *Z*‐scores > 1.65 or < −1.65 for each brain region were coded green (hypermetabolic) or red (hypometabolic) on a gradient, so darker colors represented greater absolute values. This allowed for a visual comparison of abnormalities between patients. Qualitative data about body PET findings, noting increased region(s) or muscle(s) with increased uptake, and grading of the abnormality as mild, moderate, or marked. These data were reviewed and compared between groups by SPSD phenotype.

Between groups, statistical analysis by SPSD phenotype, was completed using the Fisher exact test for categorical variables (age, sex, race, ethnicity, abnormal examination findings, medication use, presence of abnormal PET, mRS) and Kruskal Wallis with correction for multiple comparisons for continuous variables (timed 25‐foot walk at time of first PET, GAD65 titer). To evaluate if GAD65 titer was related to the presence of abnormal brain or body PET findings, patients seropositive for GAD65 were separated into quartiles based on titer, and between‐group differences were evaluated. A *p* value cutoff of 0.05 was used. Analyses were completed using SPSS version 29.0.

## 3 Results

### 3.1 Demographics and Characterization of Study Participants

Eighteen patients met inclusion criteria, including 10 SPS plus, 6 classic SPS, and 2 PERM patients. The mean age of symptom onset for the cohort was 50.7 (SD 8.5 years) years; the majority (77.8%) were female and Black/African American (50.0%), while 44.4% were White and 1 (5.6%) identified as Hispanic (Table 1). Thirteen participants (72.2%) were seropositive for anti‐GAD65, 1 (5.6%) had anti‐amphiphysin, and 4 (22.2%) tested positive for anti‐glycine receptor antibodies. Titers varied among the 13 patients with GAD65 seropositivity, with a range from 409 to 1,218,000 IU/mL and a median of 256,000 IU/mL.

**TABLE 1:** Summary of demographic and clinical characteristics for the Stiff Person Syndrome Spectrum Disorders Cohort.

Characteristics	All	Classic SPS (*n* = 6)	SPS plus (*n* = 10)	PERM (*n* = 2)
Mean Age at Symptom Onset, years	50.7	41.5	58.1	41.1
Female, *n* (%)	14 (77.8%)	4 (66.7%)	9 (90.0%)	1 (50.0%)
Race, *n* (%)				
White	8 (44.4%)	5 (83.3%)	4 (40.0%)	0 (0.0%)
Black/African American	9 (50.0%)	1 (16.7%)	6 (60.0%)	2 (100.0%)
Hispanic	1 (5.6%)	0 (0.0%)	1 (10.0%)	0 (0.0%)
Antibody type, *n* (%)				
GAD65 seropositive	13 (72.2%)	3 (50.0%)	9 (90.0%)	1 (50.0%)
Glycine receptor	4 (22.2%)	2 (33.3%)	1 (10.0%)	1 (50.0%)
Amphiphysin	1 (5.6%)	1 (16.7%)	0 (0.0%)	0 (0.0%)
CSF GAD65 positive	10^a^ (55.6%)	3 (30.0%)	7 (70.0%)	0 (0.0%)

^a^ CSF GAD65 titers were obtained in 11 of the 18 patients.

Between‐group statistical analyses based on SPSD phenotype revealed a statistically significant difference in race between groups, with 6 Black patients in the SPS plus (60.0%) and 2 in the PERM (100.0%) groups compared to classic SPS, in which patients were predominantly White (83.3%) (*p* < 0.05). There were no statistically significant differences in age, sex, ethnicity, GAD65 titer, or clinical characteristics including timed 25‐foot walk, mRS, or immune therapies at the time of the clinical visit nearest to the first PET scan. The only clinical examination finding that significantly differed between the groups around the time of the first PET scan was abnormal cranial nerve findings for the SPS plus patients (*p* < 0.05), consistent with the brainstem/cerebellar findings typical in this patient population.

All 18 patients had two or more whole‐body PET scans. Eight patients had two or more dedicated brain PET scans that were of sufficient quality to obtain data using NeuroQ.

### 3.2 Brain FDG‐PET Metabolism Patterns

**FIGURE 1:**
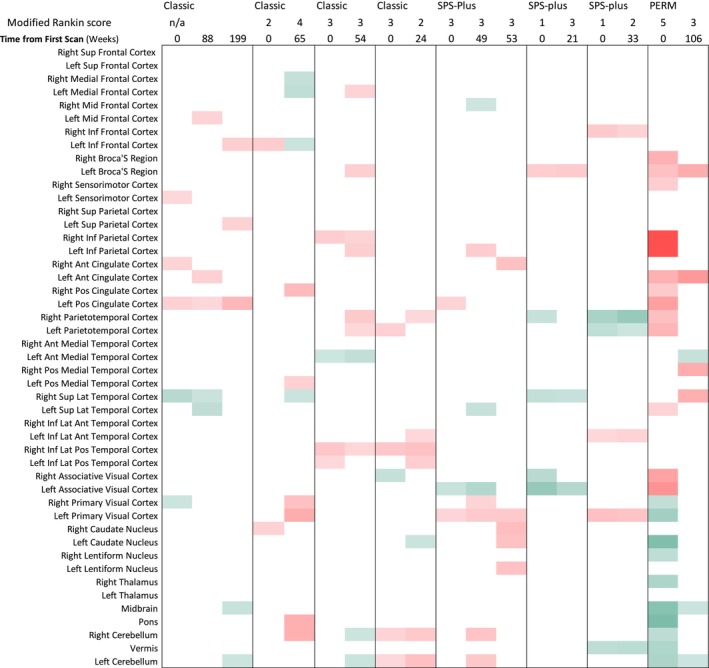
Heatmap illustrating the initial and follow‐up brain PET scans of eight patients diagnosed with either classic SPS, SPS plus, or PERM. Each patient's modified Rankin scale (mRS) is indicated alongside the time interval from the first scan, measured in weeks. Brain regions are color‐coded, with green representing hypermetabolic activity and red indicating hypometabolic activity. The heatmap provides a visual representation of metabolic changes over time, highlighting the clinical variability among patients.

Using the NeuroQ software, we observed at least two regions of abnormality in all 8 patients across multiple brain scans; however, only 8 of 18 total scans (44.4%) were read as abnormal when evaluated by a radiologist in the clinical domain. Regions of abnormality tended to differ between patients, although similar regions tended to stay abnormal for any given patient longitudinally (Figure 1). Across the entire cohort, we generally observed hypermetabolic activity in the brainstem, particularly in the midbrain and pons, as well as the cerebellum. Conversely, hypometabolic activity was observed in the frontal, temporal, parietal, and occipital lobes. However, there did not appear to be a discernible pattern by SPSD phenotype.

Four out of the seven (57%) patients receiving immune therapies and six out of the eleven patients (54.5%) not on such therapies exhibited normal brain PET imaging at their initial scan, although all patients had abnormal body PET results at that time.

**FIGURE 2:**
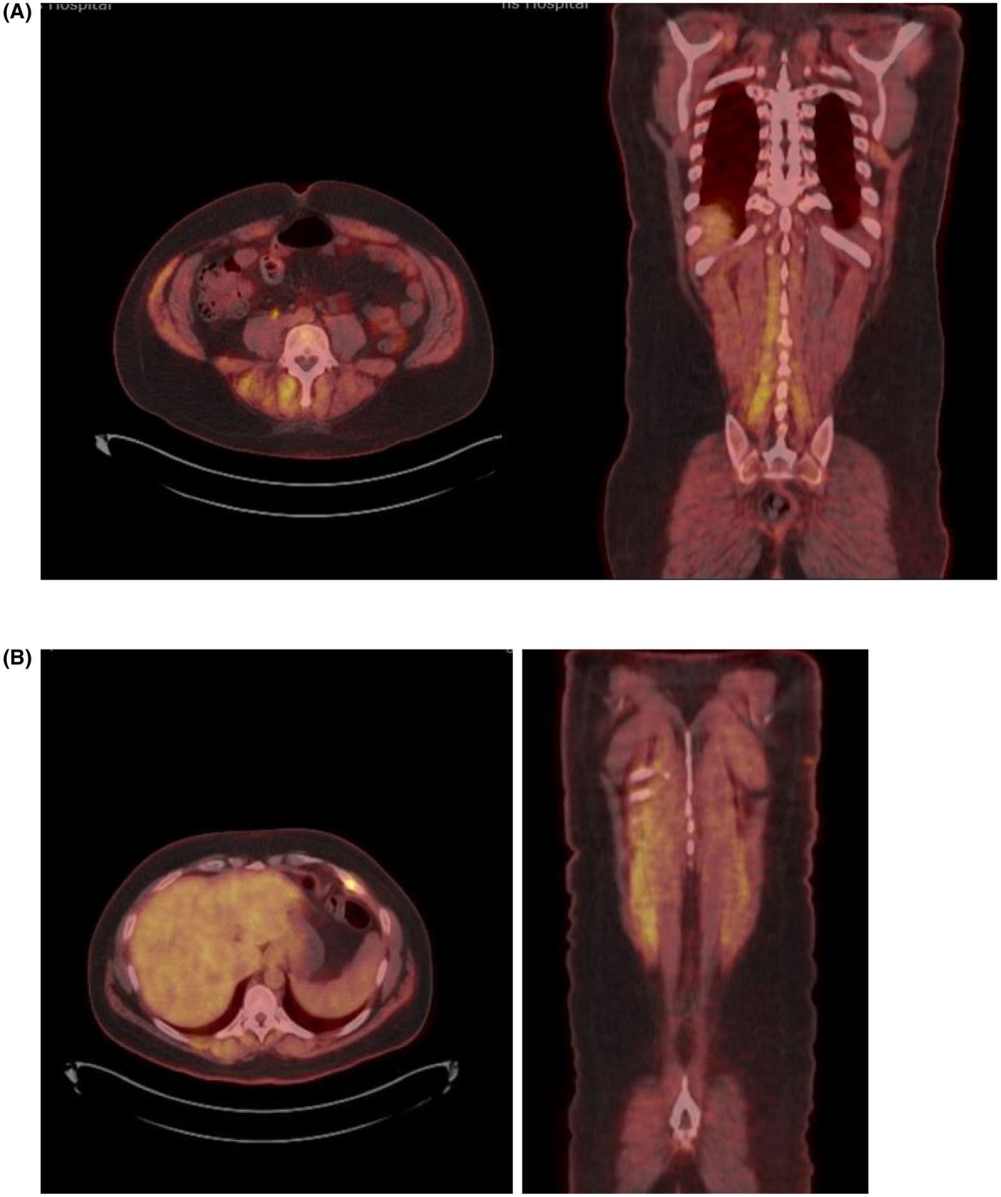
Positron emission tomography (PET) imaging illustrating abnormal uptake patterns in various body regions. (A) Axial image demonstrates abnormal uptake in the paraspinal muscles, complemented by a coronal view highlighting the same region. (B) Axial image demonstrates abnormal muscle uptake in the paraspinal muscles and posterior torso muscles on coronal imaging.

Follow‐up brain PET scans worsened over time (i.e., more abnormal regions) in 5 patients, all of whom had no addition of immunotherapy between scans. In contrast, the 2 patients with improvements in brain PET abnormalities had immune therapy added to their treatment regimen between scans. One of these patients was a PERM patient who was in the intensive care unit with a mRS of 5 at the time of the first scan and was treated with various immune therapies prior to the follow‐up PET scan, including plasmapheresis (PLEX), intravenous immune globulin (IVIG), cyclophosphamide, and rituximab (Tables 2, 3). At the time of this patient's second scan, there were notable improved clinical (improved mRS to 3) and brain imaging findings. This was the only patient with an improved mRS at the time of the follow‐up PET scan. The remaining 2 patients had stable abnormalities, only one of which had the addition of immunotherapy between scans. GAD65 titer did not correlate with the presence of abnormalities on brain PET (Figure 2).

**TABLE 2:** The various immune treatments and symptomatic therapies administered to patients at the time of first PET scan, categorized by the three subtypes of Stiff Person Syndrome Spectrum Disorders (SPSD): Classic SPS, SPS plus, and progressive encephalomyelitis with rigidity and myoclonus (PERM).

	All	Classic SPS (*n* = 6)	SPS plus (*n* = 10)	PERM (*n* = 2)
Immune treatments				
IVIG	6 (33.3%)	2 (33.3%)	4 (40.0%)	0 (0.0%)
PLEX	3 (16.7%)	1 (16.7%)	1 (10.0%)	1 (50.0%)
Rituximab	1 (5.6%)	0 (0.0%)	0 (0.0%)	1 (50.0%)
MMF	1 (5.6%)	1 (16.7%)	0 (0.0%)	0 (0.0%)
None	11 (61.1%)	4 (66.7%)	6 (60.0%)	1 (50.0%)
Symptomatic treatments				
BZD	12 (66.7%)	5 (83.3%)	7 (70.0%)	0 (0.0%)
Baclofen	7 (38.9%)	3 (50.0%)	4 (40.0%)	0 (0.0%)
Botox	1 (5.6%)	0 (0.0%)	1 (10.0%)	0 (0.0%)

**TABLE 3:** The timing of immunotherapy relative to PET imaging.

Patient	Immunotherapy (Start date)	Initial PET date	Follow‐up PET date
1	IVIG (3/2023)	2/21/2022	3/6/2023
2	IVIG (2/2006), Mycophenolate (6/2011)	6/2/2010	2/6/2012
3	IVIG (1/2022)	9/9/2021	3/17/2023
4	IVIG (2/2022)	12/16/2021	8/9/2022
5	IVIG (12/2021)	8/13/2021	2/14/2023
6	IVIG (2/2022)	1/22/2021	6/30/2022
7	IVIG (6/2022)	3/15/2021	3/17/2023
8	PLEX, IVIG, Cyclophosphamide (7/2019)	8/16/2019	8/27/2021
9	IVIG (4/2015)	4/20/2015	11/1/2017
10	Rituximab (8/2014)	10/23/2014	12/5/2018
11	IVIG (5/2021)	12/22/2020	4/14/2023

### 3.3 Skeletal Muscle FDG‐PET Metabolism Patterns

**FIGURE 3:**
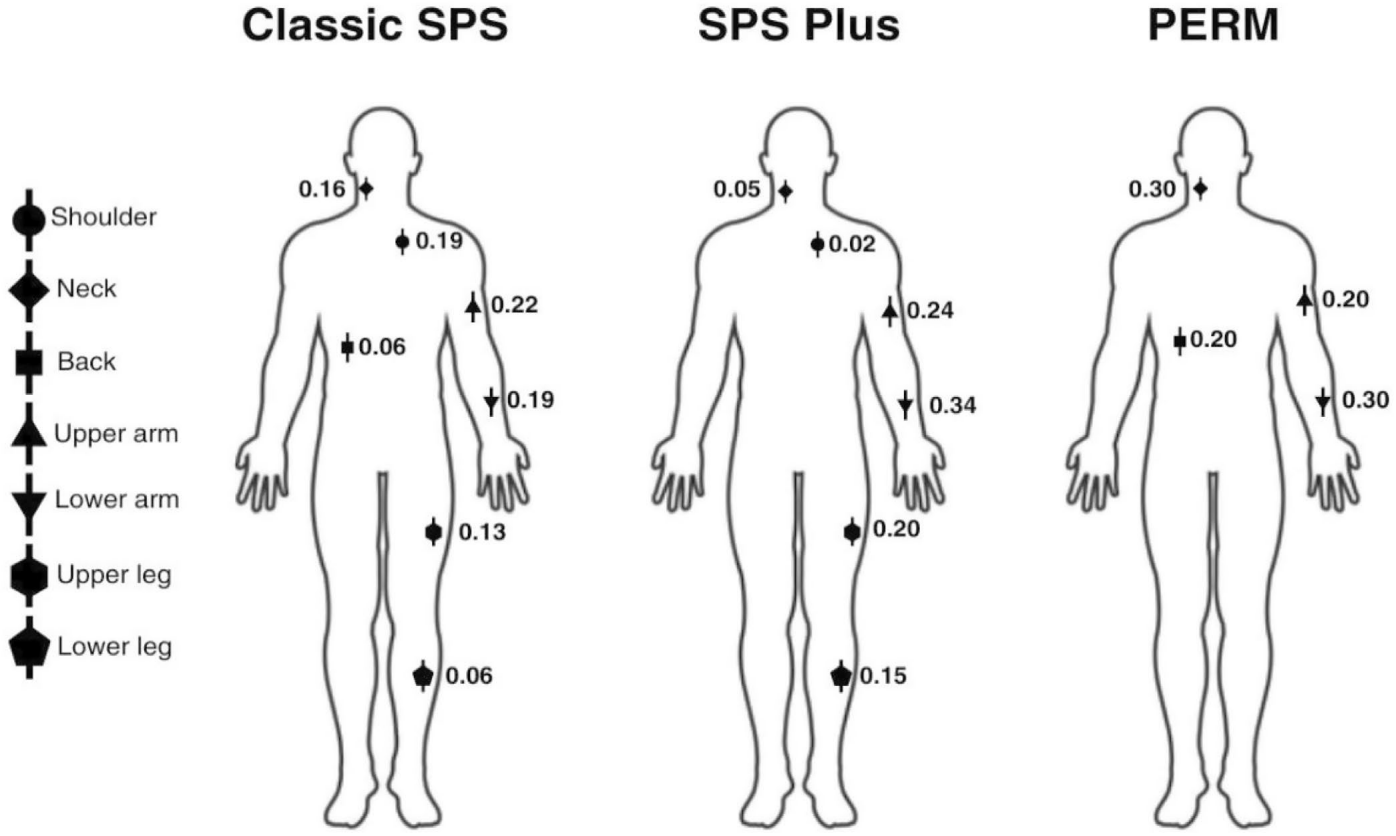
Illustration depicting the proportions of muscle involvement (0 meaning no involvement and 1 meaning exclusively involved in one muscle group) characterized by abnormal muscle uptake observed in body PET scans of patients diagnosed with classic SPS, SPS plus, and PERM. The figure highlights the specific regions of muscle involvement, as denoted by the symbols on the left side. Each symbol corresponds to distinct muscle groups that exhibit abnormal uptake, underscoring the variability in muscle involvement among the patient populations.

Among the 18 patients in this study, there were a total of 42 body PET scans; 5 patients had 3 total scans, 1 patient had 4 scans, and the remaining 12 patients had 2 scans. The forearm muscles emerged as the most commonly involved region of involvement (11 patients), followed by the lower extremities, upper arm, neck strap muscles, and shoulders. Specifically, in patients diagnosed with classic SPS, upper and lower arm muscles were the most frequently affected, followed by the neck and shoulder regions. Similarly, 7 patients with SPS plus showed a marked abnormality in forearm muscle uptake, which was by far the most pronounced, followed by legs. Both classic SPS and SPS plus phenotypes had similar abnormal muscle uptake on PET. One patient diagnosed with PERM exhibited minimal abnormal muscle uptake, with the forearm/lower arm being the area of greatest involvement (Figure 3). 

Longitudinally, it became evident that for any given patient, the abnormal uptake in skeletal muscles observed on body PET images remained abnormal on follow‐up scans. There was no clear association between patients being started on an immunotherapy and change or stability in body PET abnormalities.

When comparing the nuclear medicine review of the skeletal muscle on PET scans to the clinical radiology report of the skeletal muscle for the same scan, there were discrepancies in 20 (47.6%) scans. In all of these cases, there was evidence of increased skeletal muscle FDG uptake on the scan, but the clinical report only specified the absence of malignancy, without commenting on any other abnormalities that may have been present. GAD65 titer did not correlate with the presence of abnormalities on body PET.

## 4 Discussion

The present study describes the longitudinal changes in brain and skeletal muscle FDG uptake on PET scan in a cohort of patients with SPSD. Consistent with our group's prior work, all patients demonstrated abnormal brain PET findings using NeuroQ, and many had increased muscle uptake on their initial body PET scan. These findings suggest that in the appropriate clinical picture, abnormalities on PET may represent an imaging biomarker that can help support a SPSD diagnosis. Generally, we observed that the abnormalities in the brain and skeletal muscle varied between patients, but for any given patient, the same areas tended to remain abnormal over time, suggesting findings are specific to the individual. Furthermore, the initiation of immune therapy between scans may correlate with stabilization or improvement of abnormalities on brain PET, although this was not evident on body PET. We hypothesized that each SPSD phenotype would demonstrate different patterns of PET abnormality, corresponding to the predominant symptoms of each phenotype. For example, we expected SPS plus patients to have more brainstem and cerebellar abnormalities on brain PET, and expected classic SPS patients to demonstrate more extensive abnormal skeletal muscle uptake on body PET.

While there were no distinct patterns of brain hypermetabolism or hypometabolism identified by SPSD phenotype, we observed that in many patients, the infratentorial regions appeared predominantly hypermetabolic. In contrast, the frontal, temporal, parietal, and occipital lobes tended to be hypometabolic. Although most of the patients in this cohort were on symptomatic therapies, including benzodiazepines, which have the potential to influence brain metabolism on PET, we would expect these changes to be relatively symmetric, and the abnormalities we observed in our cohort were largely asymmetric. This supports the idea that the abnormalities may be from the underlying pathophysiology of SPSD and not a medication effect [10, 17].

Contrary to our hypothesis, there were no clear patterns of brain PET abnormalities when comparing different SPSD phenotypes. In fact, patients with all SPSD phenotypes had abnormalities in the brainstem and cerebellum, suggesting that the relationship between clinical phenotype and brain dysmetabolism may not be straightforward. Moreover, this highlights the strong connection(s) between the cerebrum and posterior fossa structures. A possible explanation for this finding may be compensatory mechanisms with different structures based on metabolic pattern (e.g., frontal lobe hypometabolic activity and hypermetabolic infratentorial regions). Additionally, the cerebellum's association with hypermetabolic activity may stem from its connections with the frontal lobe, which could influence muscle movement through downregulation mechanisms. Understanding these neural connections may provide insights into the observed metabolic patterns.

The majority of patients who were not on immune therapy at the time of the first scan and who did not receive immunotherapies between scans exhibited worsened brain PET findings at follow‐up (i.e., increased number of brain regions involved), potentially mirroring disease progression in the absence of therapeutic intervention. PET improvement (i.e., decreased number of brain regions involved) or stability (i.e., no change in the number of brain regions involved) with the introduction of immunotherapy underscores the potential impact of immunotherapy on the pathology within the central nervous system for people with SPSD, suggesting that such interventions may mitigate disease progression. However, prospective, randomized controlled trials would be required to investigate this further.

Most SPSD patients in our cohort had increased FDG skeletal muscle uptake on body PET, with the notable exceptions of three patients with SPS plus and one patient with a sensory neuronopathy at the time of first PET scan, who eventually developed signs and symptoms of classic SPS in the presence of amphiphysin antibodies. All patients with a classic SPS phenotype at the time of PET scan exhibited at least one region of increased skeletal muscle FDG uptake. In contrast, only a portion of SPS plus patients had abnormal skeletal muscle uptake, which might be explained by the predominant brainstem and cerebellar symptoms in this phenotype, as compared to the muscle stiffness/spasms predominance in classic SPS.

One of the PERM patients in our cohort showed minimal abnormal muscle uptake, with forearms being the most commonly affected area. In contrast, the brain PET was floridly abnormal, suggesting that the different subtypes of SPSD are heterogeneous in presentation and can be confounded by symptomatic and immune therapy use. However, this finding could also be due to the fact that the patient was not experiencing significant rigidity and/or myoclonus at the time of PET imaging, in the acute setting, with many symptomatic and immune therapies being administered. On the other hand, our other PERM patient exhibited diffuse hypometabolism in the cortices and hypermetabolism in the infratentorial regions on the initial brain PET scan, while both the initial and follow‐up body PET scans were reported as normal.

Among all SPSD patients, the forearm muscles were the most commonly involved muscle group, followed by the upper arms. Abnormalities were symmetric in most cases. Proximal leg (thigh) muscle stiffness/spasms are often seen in SPSD, as a common pattern of involvement, along with involvement of torso musculature and hip flexors, limiting patients’ ability to bend at the hip and ambulate. The involvement of forearm musculature in many patients was an unexpected finding, as the lower limbs are typically more involved in SPSD by history and on exam. One possible explanation for the forearm hypermetabolism is sustained muscle activity from patients gripping the PET/CT table during image acquisition. In addition to the forearms and legs, we noted considerable involvement of the shoulders and neck strap muscles in both classic SPS and SPS plus phenotypes. The increased muscle activity in the neck region is consistent with the axial rigidity typical of SPSD and can often be detected on examination, but may be influenced by factors such as muscle use, posture, or compensatory mechanisms in response to the disease.

Given the high frequency of increased FDG uptake in forearm and leg muscles, we hypothesized that individuals using assistive devices for mobility due to leg stiffness might exhibit increased physiological uptake in both regions simultaneously on body PET imaging due to engagement of those muscles while ambulating. Seven patients showed involvement of the forearm muscles in body PET imaging during either the initial or follow‐up scans. In the initial body PET scan, only 3 out of 7 patients were using assistive devices, such as walkers or canes. In the follow‐up body PET imaging, 5 out of 7 patients were using assistive devices at the time of imaging. It is possible that symptomatic therapies impacted these findings or the qualitative assessments.

Discrepancies between the clinical radiology report and the results from NeuroQ and independent nuclear medicine review focused on the skeletal muscle detected discrepancies in 44.4% of the brain PET scans and 47.6% of the body PET scans. In all of these cases, abnormalities were detected when the scans were interpreted as unremarkable in the clinical report. Notably, radiologists are typically reviewing the scans with the purpose of ruling out a malignancy, and this discrepancy highlights why it is important to specify an interest in evaluating for increased FDG uptake in the brain and skeletal muscle when an SPSD diagnosis is being evaluated.

This study has several limitations that must be acknowledged to contextualize our findings accurately. First, the absence of healthy control participants in patients with body PET imaging is a limitation. Without this comparison, it is challenging to determine which observed abnormalities in body PET imaging may be inherent to the disease versus those that could be considered physiologic variations, especially in regions of the body that are not expected to be involved in SPSD. While the lack of healthy controls limits direct comparisons, it underscores the unique characteristics of patients with SPSD and presents an opportunity for future studies to incorporate healthy controls to establish baseline metrics for comparison. Second, the relatively small cohort size, particularly within each subtype of SPS, limits the statistical power and significance of our findings. However, this is the largest cohort to date looking at longitudinal PET scan assessments of these patients. Variability in the timing of these scans, especially relative to patients’ symptomatic therapies and whether they were having spasms at the time of the scan, could introduce confounding factors that may affect brain and muscle metabolic activity and PET results. In addition, body PET observations were qualitative in nature and were reported by body region, rather than individual muscles. One consideration is that patients may not have been experiencing spasms at the time of imaging, which could influence the observed PET findings. We were not able to capture the timing of patients’ symptomatic medications in relation to the PET study, which could have influenced the body findings, and future studies should aim to capture these data as well as whether spasms were occurring during the scan.

Future studies are needed to investigate the utility of brain and body PET as a biomarker for both the diagnosis of SPSD and monitoring treatment response. Future randomized controlled trials should standardize the timing of PET imaging relative to immune and symptomatic therapy administration for baseline and follow‐up scans, which would allow for a more accurate comparison of scans between patients. The incorporation of advanced technologies, such as artificial intelligence and machine learning, could aid in future investigations, particularly in identifying subtle PET abnormalities and differentiating pathologic and physiologic uptake. These tools may provide deeper insights into the nuances of body region(s) involvement across different patient profiles.

## 5 Conclusion

Our study contributes to the understanding of brain and skeletal muscle metabolism in patients with SPSD over time, highlighting the complexity of metabolic activity and its variability across different phenotypes. Our analysis of body PET findings in patients with SPSD highlights the complexity of muscle involvement over time and the interplay between central and peripheral pathophysiological processes. Further studies, ideally with larger cohorts and control groups, are warranted to delineate the brain and body PET patterns and their clinical implications more clearly.

## Author Contributions

M.M.Q. assisted with data analysis, drafted the initial manuscript, and edited the final version. S.N.R. collected and analyzed the data including PET imaging and drafted the initial manuscript. M.S.S. and L.B.S. contributed to data collection and interpretation of PET images for patients with SPSD. S.D.N. served as corresponding author, supervised and conceptualized the study, reviewed and edited the first and final version of the manuscript. All authors reviewed the manuscript and approved the final version for submission.

## Conflicts of Interest

The authors declare no conflicts of interest.

## Data Availability

The data that support the findings of this study are available on reasonable request from the corresponding author. The data are not publicly available due to privacy or ethical restrictions.
